# Identification of immune associated potential molecular targets in proliferative diabetic retinopathy

**DOI:** 10.1186/s12886-023-02774-y

**Published:** 2023-01-19

**Authors:** Ying Gao, Min Xue, Bing Dai, Yun Tang, Jingyu Liu, Changlin Zhao, Hu Meng, Feng Yan, Xiaomin Zhu, Yan Lu, Yirui Ge

**Affiliations:** 1 Department of Ophthalmology, Affilia Jinling Hospital, School of Medicine, Nanjing University, Nanjing, Jiangsu Province China; 2 Department of Ophthalmology, Anhui NO.2 Provincial People’s Hospital, Hefei, Anhui China; 3 Department of Vascular Surgery, Tianjin Hospital, Tianjin, China

**Keywords:** Proliferative diabetic retinopathy, Neovascular membrane, Immune, Inflammation, Genes, Biomarkers

## Abstract

**Background:**

Diabetic retinopathy (DR) is one of the most common microvascular complications of diabetes and causes of blindness in developed countries. Our study was designed to identify immune-related genes involved in the progression of proliferative diabetic retinopathy (PDR).

**Methods:**

The “GSE102485” dataset of neovascular membrane samples (NVMs) from type 1 and 2 diabetes mellitus patients was downloaded from the Gene Expression Omnibus database. Functional enrichment analyses, protein–protein interaction network (PPI) construction, and module analysis of immune pathways in NVMs and controls were conducted via Gene Set Enrichment Analysis and Metascape.

**Results:**

The significantly upregulated hallmark gene sets in DR2 and DR1 groups were involved in five immune pathways. Only CCR4, CXCR6, C3AR1, LPAR1, C5AR1, and P2RY14 were not previously reported in the context of PDR molecular pathophysiology. Except for P2RY14, all of the above were upregulated in retinal samples from experimental diabetes mouse models and human retina microvascular endothelial cells (HRMECs) treated with high glucose (HG) by quantitative Real Time Polymerase Chain Reaction (qRT-PCR).

**Conclusion:**

The genes identified herein provide insight into immune-related differential gene expression during DR progression.

## Background

Diabetic retinopathy (DR), resulting from chronic hyperglycemia, is one of the most common microvascular complications of diabetes and causes of blindness among adults aged between 20 and 74 in developed countries [1, 2]. Depending on the degree of related ischemic injury and microvascular lesions, DR can be divided into two phases, non-PDR (proliferative diabetic retinopathy) and PDR [3]. DR is recognized as a microvascular, inflammatory, and neurodegenerative complication of diabetes [4], which can be triggered by mitochondrial damage, endoplasmic reticulum stress, and oxidative stress, among others [5]. However, the pathogenesis of DR has not been fully elucidated. Inflammation and angiogenesis play key roles during DR pathogenesis [6]. Thus, the morphological and molecular alterations in DR caused by these two processes are receiving great attention, becoming the focus of extensive research [7, 8].

Chronic low-grade inflammation is present during both the early and advanced stages of DR, eventually resulting in retinal vasculopathy, which is characterized by increased retinal vascular permeability and neovascularization [9, 10]. Inflammation is undoubtedly implicated in the dysregulated pathological angiogenesis during early and advanced stages of DR [11]. Neovascularization and inflammation share several common mediators and signaling pathways [11, 12]. Inflammatory responses drive angiogenic processes through the production of pro-angiogenic cytokines and growth factors [11–13].

Shao et al. identified a subset of differentially expressed genes (DEGs) from both active and inactive fibrovascular membranes (FVMs) with normal retinas, which were enriched for angiogenic factors [hypoxia inducible factor-1 subunit alpha (HIF-1α) and placental growth factor (PGF)] [14]. Pathological secretion of vascular endothelial growth factor A was shown to promote the expression of pro-angiogenic transcription factors and growth factors, which in turn induced retinal neovascularization [15]. In the present study, we first analyzed an RNA-seq dataset of NVMs from PDR patients from the GEO database. We identified genes associated with the immune system in order to elucidate the role of inflammatory processes during DR pathogenesis and identify novel diagnostic and therapeutic markers for DR.

## Methods

### Dataset

The clinical sample dataset “GSE102485” was downloaded from GEO (http://www.ncbi.nlm.nih.gov/geo/). Twenty-five samples from “GSE102485” were analyzed, including 19 samples of type 2 DR, three from type 1 DR, and three normal retina samples.

### Data grouping

The 25 samples were of NVMs from PDR and were divided into three paired groups: DR2 group (type 2 PDR and normal retina), DR1 group (type 1 PDR and normal retina), and DR2 VS DR1 group (type 2 PDR and type 1 PDR).

### Gene set enrichment analysis (GSEA)

The DR2 and the DR1 groups were respectively subjected to GSEA. GSEA was implemented to detect the enriched gene sets for the two paired groups respectively, so as to identify the potential hallmarks of DR. The annotated gene sets of “h.all.v7.2.symbols.gmt” in the Molecular Signatures Database (MSigDB) were selected in GSEA version 4.0.3, and 1000 times of permutations were conducted. Collapse dataset of gene symbols was termed as “no-Collapse”, and the permutation type was “phenotype”. The cut-off criteria for GSEA were as follows: normalized enrichment scores (NES) > 1.0; false discovery rate (FDR) *q*< 0.25; nominal *p* < 0.05. The minimum number of 15 genes and maximum 500 genes were set by default. All significantly enriched immune-related hallmark gene sets were collected and displayed via enrichment plots. Immune-related hub genes in the DR2 and DR1 groups were then identified using Kyoto Encyclopedia of Genes and Genomes (KEGG) and Gene Ontology (GO) enrichment analyses.

### Metascape hub gene analysis

Metascape (http://metascape.org) is a free website analysis tool for gene function annotation and pathway enrichment analysis [16]. We used it for process enrichment as well as pathway and protein–protein interaction (PPI) enrichment analysis of immune-related hub gene sets. The highest-scoring genes in both groups were presented in Venn diagrams (http://bioinformatics.psb.ugent.be/webtools/Venn/).

### Animal model and HRMECs culture

Adult C57Bl/6 J mice (male, 8 weeks old, 10–22 g), provided by the Laboratory Animal Center, Nanjing Medical University, were used in this study. Animal experiments were performed in accordance with the criteria of the National Institutes of Health guide for the care and use of laboratory animals as well as the ARRIVE guidelines. The Ethics Committee of the Affiliated Jinling Hospital of Nanjing University approved the study protocol (2021JLHDWLS-007). After housing for 24–36 h, mice were intraperitoneally administered streptozotocin (Sigma, 0.1 mg per 10 g of bodyweight) in citrate saline. Mice with blood glucose levels over 16.7 mmol/L for two consecutive weeks were considered diabetic. After 4 months, diabetic and age-matched non-diabetic mice were sacrificed via excessive intraperitoneal injection of the mixture of ketamine and xylazine, both eyes of each mouse were enucleated, and retinas were detached. Retinal samples were then moved and subjected to cryopreservation. HRMECs were purchased from American Type Culture Collection (ATCC, U.S.A.). HRMECs were cultured in endothelial cell medium (ECM, Gibco) supplemented with 10% fetal bovine serum (FBS, Gibco, U.S.A.), penicillin and streptomycin (100 U/ml) at 37 °C under 5% CO_2_. To detect the effect of glucose on HRMECs, the medium was further supplemented with high concentration of glucose (35.5 mM; HG) and normal concentration of glucose (5.5 mM; NG) for 24 h,respectively.

### RNA isolation and qRT-PCR

Total RNA was extracted from the retinal tissue of diabetic retinopathy mouse models using TRIzol reagent (Invitrogen). Total RNA was then reverse-transcribed using a PrimeScript RT reagent Kit (Takara). Glyceraldehyde-3-phosphate dehydrogenase (GAPDH) was detected as an internal control.

Total RNA of HRMECs was extracted using TRIzol reagent (Invitrogen), and was then reverse-transcribed using a PrimeScript RT reagent Kit (Takara). The cDNA was used as the template for qRT-PCR. respectively. Actin beta (ACTB) was detected as an internal control.

The reaction mixture (20 μL) contained 1 μL cDNA template, 2 μL (10 μM) each of sense and antisense primers (designed by Primer6, Table 1), 0.1% DEPC 7 μL, and 10 μL Real-time PCR Master Mix (SYBR Green). qRT-PCR was performed on an ABI Step one plus qRT-PCR system (Applied Biosystems). qRT-PCR was performed in duplo for each sample, and dissociation curves were used to estimate the specificity of qRT-PCR products.

**Table 1:** Gene primer information of diabetic retinopathy mouse models and HRMECs

Gene	Forward Primer	Reverse Primer
Gene primer information of diabetic retinopathy mouse models		
C5ar1	CATACCTGCGGATGGCATTCA	GGAACACCACCGAGTAGATGAT
CXCR6	GAGTCAGCTCTGTACGATGGG	TCCTTGAACTTTAGGAAGCGTTT
C3AR1	TCGATGCTGACACCAATTCAA	TCCCAATAGACAAGTGAGACCAA
LPAR1	AGCCATGAACGAACAACAGTG	CATGATGAACACGCAAACAGTG
P2RY14	AGCAGATCATTCCCGTGTTGT	AGCCACCACTATGTTCTTGAGA
CCR4	GGAAGGTATCAAGGCATTTGGG	GTACACGTCCGTCATGGACTT
Gene primer information of HRMECs		
C5ar1	CCATCCATCCATCCATCCATCCATC	GAGGCAGGAGAATCGCTTGAACC
CXCR6	TGCCACTGCTCACCATGATTGTC	GGAACACAGCCATCACCAGGAAG
C3AR1	TGAAGATGCAGCGGACAGTGAAC	GCCAAGTGAGCCAGCGAGAAG
LPAR1	TTCAAGCGATTCTCCTGCCTAAGC	TTCAAGACCAGCCTGACCAACATG
P2RY14	TCCCTCTACACACTGCTTTGAATGC	ACTGAACAACCTGCTCCTGAATGAC
CCR4	GGCTCAAGTGATCCTCCCTCCTC	CCACCACCACACACCCAATGC

### Statistical analysis

Data were analyzed using the unpaired Student’s t-test and one-way ANOVA for multiple comparisons in Graphpad Prism 8. Data were presented as an average of 6 SEM, unless indicated otherwise. Statistical significance was set at *p* < 0.05.

## Results

### Enrichment of immune-related gene sets

**Fig. 1:**
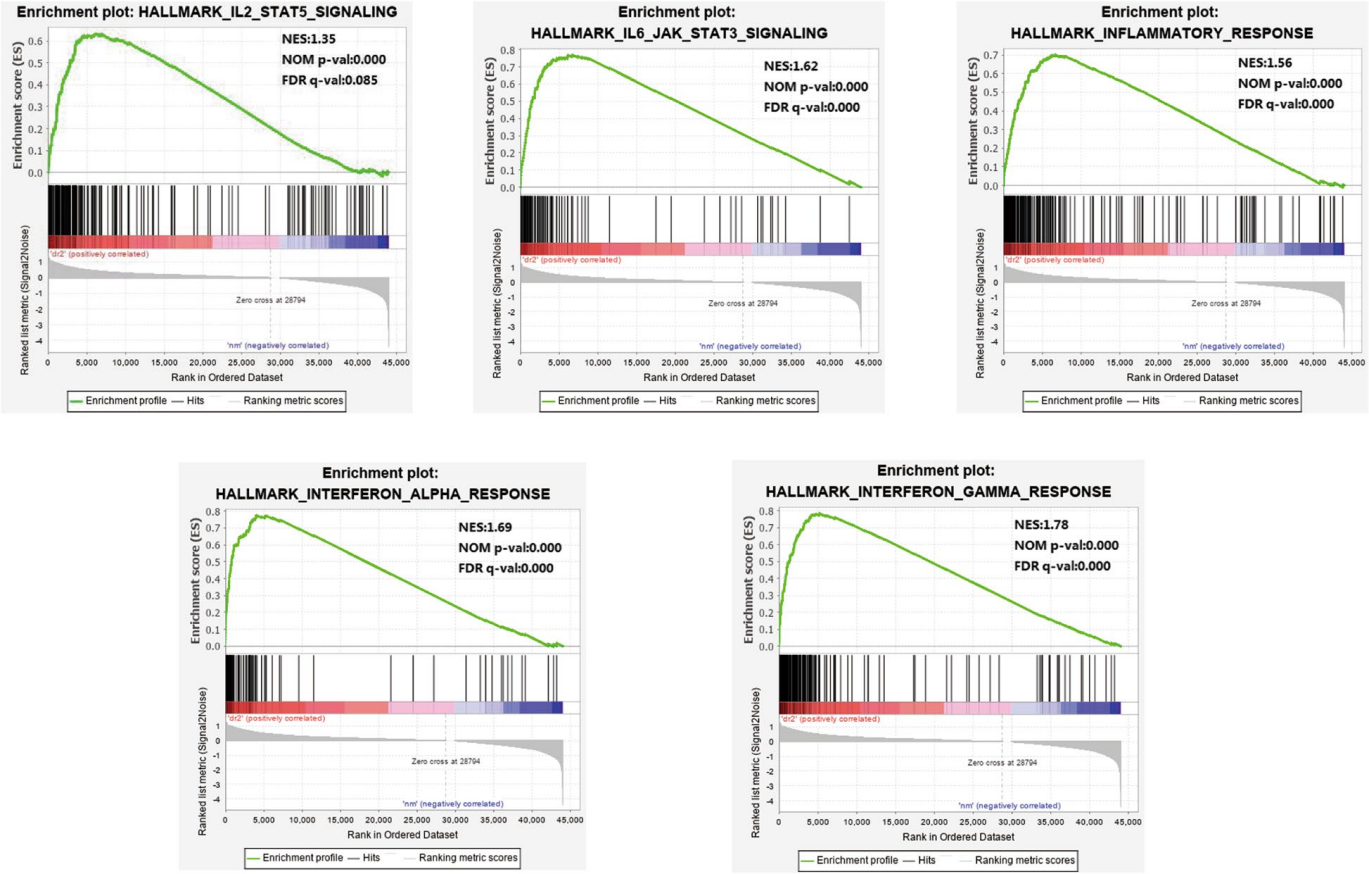
Significant immune-related gene expression in the DR2 group was analyzed via GSEA

**Fig. 2:**
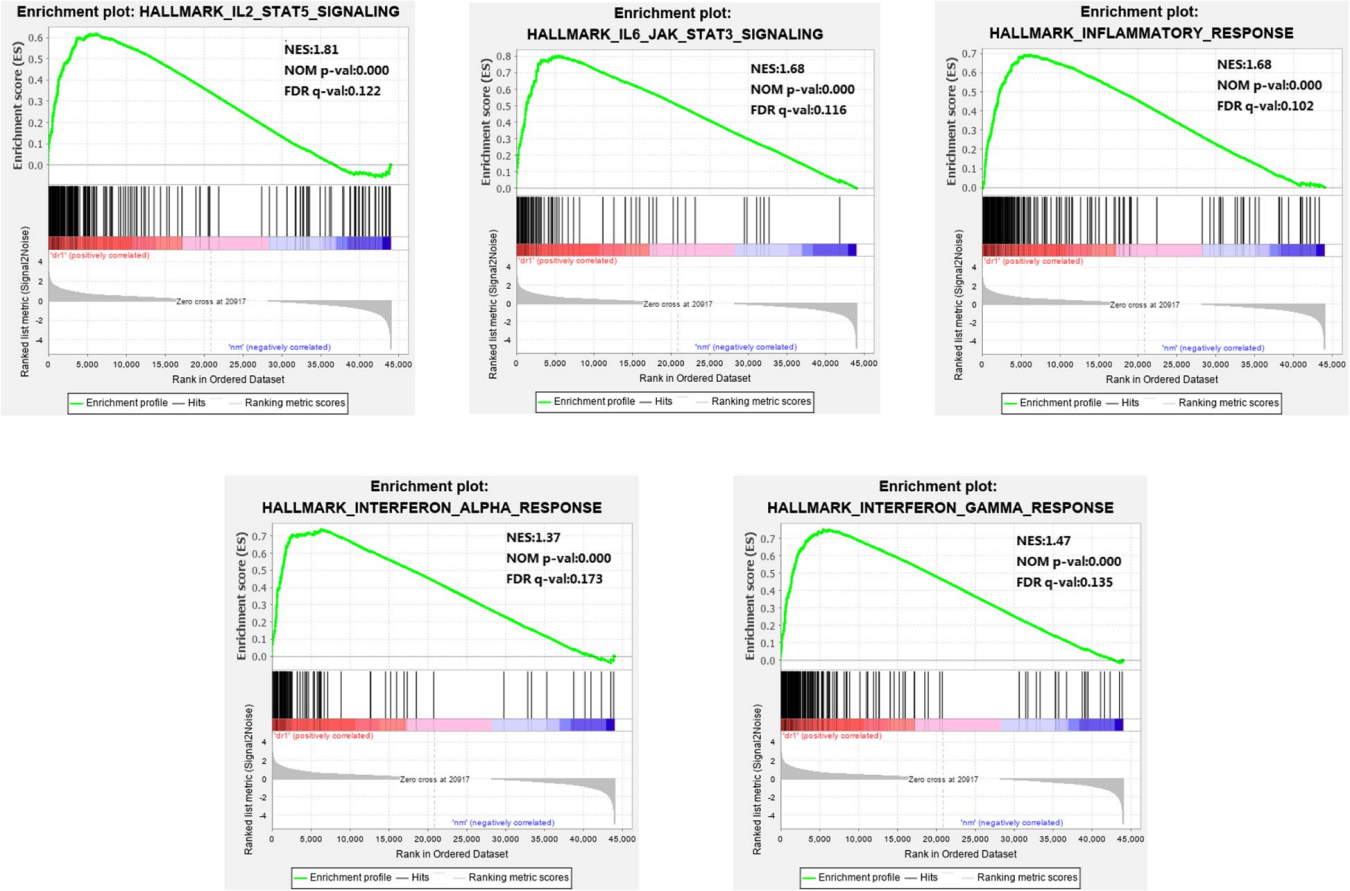
Significant immune-related gene expression in the DR1 group was analyzed via GSEA

In the DR2 phenotype, 46 out of 50 gene sets were upregulated, and 13 were significantly enriched, with a nominal *p* < 0.05, NES > 1.0, and FDR *q*<0.25. In the DR1 phenotype, 39 out of 50 gene sets were upregulated, and 11 were significantly enriched. All gene sets of the two groups are shown in Table 2 (DR2) and Table 3 (DR1). In the DR2 VS DR1 group, 28 out of 50 gene sets were upregulated in type 2 PDR, while 0 were significantly enriched, with a nominal *p* < 0.05, NES > 1.0, and FDR *q*<0.25. Twenty-two out of 50 gene sets were upregulated in type 1 PDR, and 0 were significantly enriched, with a nominal *p* < 0.05, NES > 1.0, and FDR *q*<0.25. There were no significantly enriched gene sets between the type 2 and type 1 PDR groups. Significantly upregulated immune-related hallmark gene sets in the DR2 and DR1 groups are shown in Fig. 1 (DR2) and Fig. 2 (DR1): INTERFERON_GAMMA_RESPONSE, INTERFERON_ALPHA_RESPONSE, IL6_JAK_STAT3_SIGNALING, INFLAMMATORY_RESPONSE, IL2_STAT5_SIGNALING.

**Table 2:** GSEA pathways up-regulated and down-regulated due to DR2 group

Gene sets	SIZE	NES	NOM *p*-val	FDR *q*-val
Up-regulated gene sets in DR2 group				
HALLMARK_INTERFERON_GAMMA_RESPONSE	194	1.78	0.000	0.000
HALLMARK_COAGULATION	133	1.69	0.000	0.000
HALLMARK_INTERFERON_ALPHA_RESPONSE	95	1.69	0.000	0.000
HALLMARK_EPITHELIAL_MESENCHYMAL_TRANSITION	198	1.68	0.000	0.000
HALLMARK_ANGIOGENESIS	36	1.67	0.000	0.000
HALLMARK_IL6_JAK_STAT3_SIGNALING	83	1.62	0.000	0.000
HALLMARK_INFLAMMATORY_RESPONSE	198	1.56	0.000	0.000
HALLMARK_ALLOGRAFT_REJECTION	195	1.53	0.000	0.000
HALLMARK_COMPLEMENT	198	1.38	0.000	0.042
HALLMARK_TNFA_SIGNALING_VIA_NFKB	199	1.37	0.000	0.075
HALLMARK_IL2_STAT5_SIGNALING	197	1.35	0.000	0.085
HALLMARK_XENOBIOTIC_METABOLISM	198	1.26	0.000	0.234
HALLMARK_APOPTOSIS	158	1.25	0.000	0.244
Down-regulated gene sets in the DR2 group				
HALLMARK_PANCREAS_BETA_CELLS	40	−1.91	0.000	0.000

**Table 3:** GSEA pathways up-regulated and down-regulated due to DR1 group

Gene sets	SIZE	NES	NOM *p*-val	FDR *q*-val
Up-regulated gene sets in DR1 group				
HALLMARK_COAGULATION	133	2.17	0.000	0.000
HALLMARK_IL2_STAT5_SIGNALING	197	1.81	0.000	0.122
HALLMARK_ANGIOGENESIS	36	1.69	0.000	0.135
HALLMARK_IL6_JAK_STAT3_SIGNALING	83	1.68	0.000	0.116
HALLMARK_INFLAMMATORY_RESPONSE	198	1.68	0.000	0.102
HALLMARK_EPITHELIAL_MESENCHYMAL_TRANSITION	198	1.68	0.000	0.090
HALLMARK_KRAS_SIGNALING_UP	194	1.58	0.000	0.130
HALLMARK_ALLOGRAFT_REJECTION	195	1.54	0.000	0.118
HALLMARK_INTERFERON_GAMMA_RESPONSE	194	1.47	0.000	0.135
HALLMARK_APICAL_SURFACE	44	1.44	0.000	0.150
HALLMARK_INTERFERON_ALPHA_RESPONSE	95	1.37	0.000	0.173
Down-regulated gene sets in the DR1 group				
HALLMARK_PANCREAS_BETA_CELLS	40	−1.67	0.000	0.187
HALLMARK_SPERMATOGENESIS	131	− 1.46	0.000	0.211

GO and KEGG enrichment analyses of all immune-related hub genes.

GO functional and KEGG pathway enrichment analyses of all immune-related hub genes for the DR2 and DR1 groups were carried out in Metascape.

**Fig. 3:**
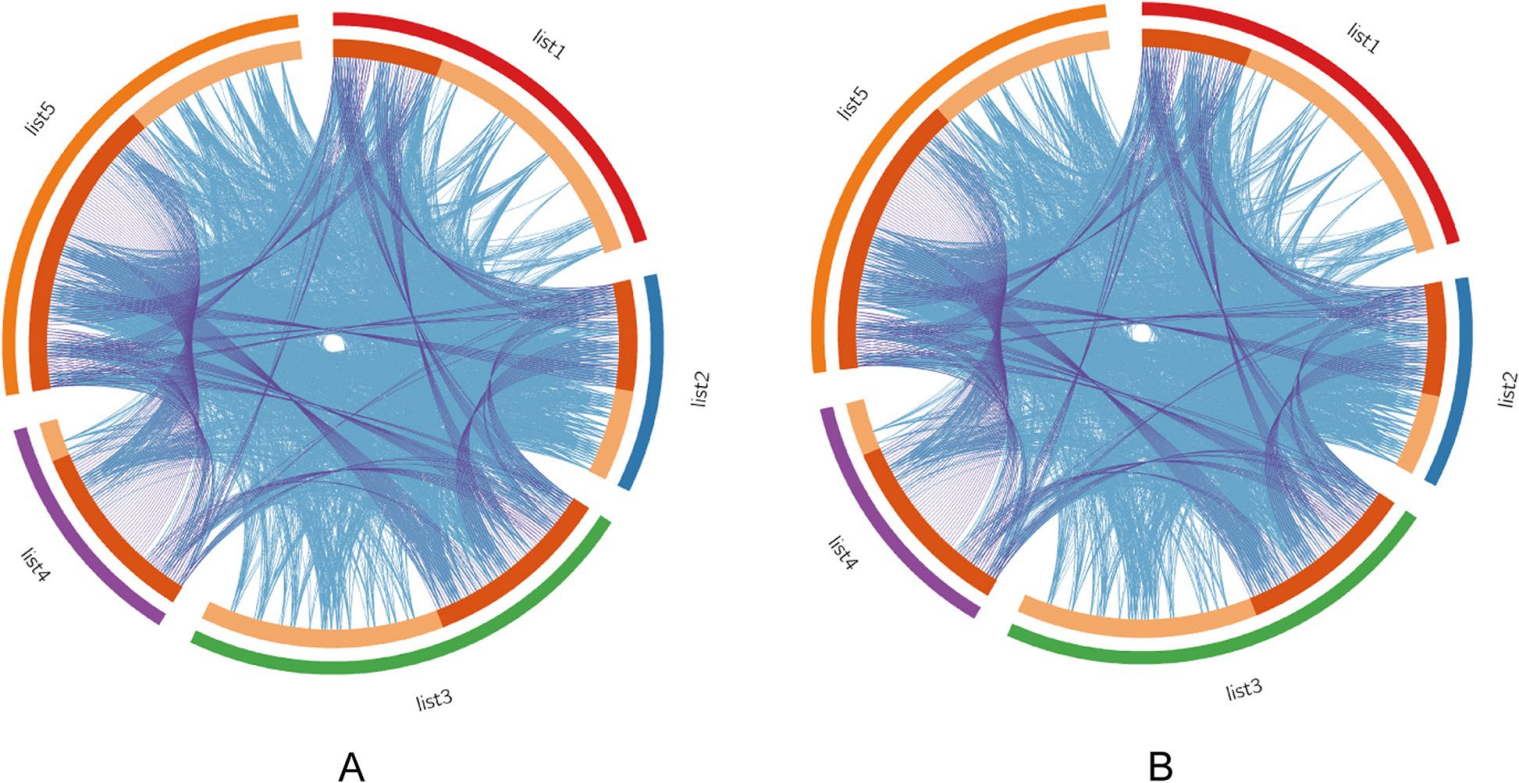
Overlaps including the shared term level, where the blue curves link genes with the same enriched ontology term for DR2 (**A**) and DR1 (**B**). The inner circles represent gene lists, where hits are shown along the arc. Multiple gene lists were colored in dark orange, while unique gene lists are shown in light orange

The overlaps in these gene lists were significantly improved by considering overlaps between genes sharing the same enriched ontology terms. Circus plots for DR2 and DR1 are shown in Fig. 3 (A, B) and Table 4.

**Table 4:** Statistics of input gene lists

Name	Description	Total	Unique
The DR2 group			
list1	HALLMARK_IL2_STAT5_SIGNALING	115	115
list2	HALLMARK_IL6_JAK_STAT3_SIGNALING	62	62
list3	HALLMARK_INFLAMMATORY_RESPONSE	133	133
list4	HALLMARK_INTERFERON_ALPHA_RESPONSE	69	69
list5	HALLMARK_INTERFERON_GAMMA_RESPONSE	149	149
The DR1 group			
list1	HALLMARK_IL2_STAT5_SIGNALING	115	115
list2	HALLMARK_IL6_JAK_STAT3_SIGNALING	58	58
list3	HALLMARK_INFLAMMATORY_RESPONSE	125	125
list4	HALLMARK_INTERFERON_ALPHA_RESPONSE	72	72
list5	HALLMARK_INTERFERON_GAMMA_RESPONSE	141	141

**Fig. 4:**
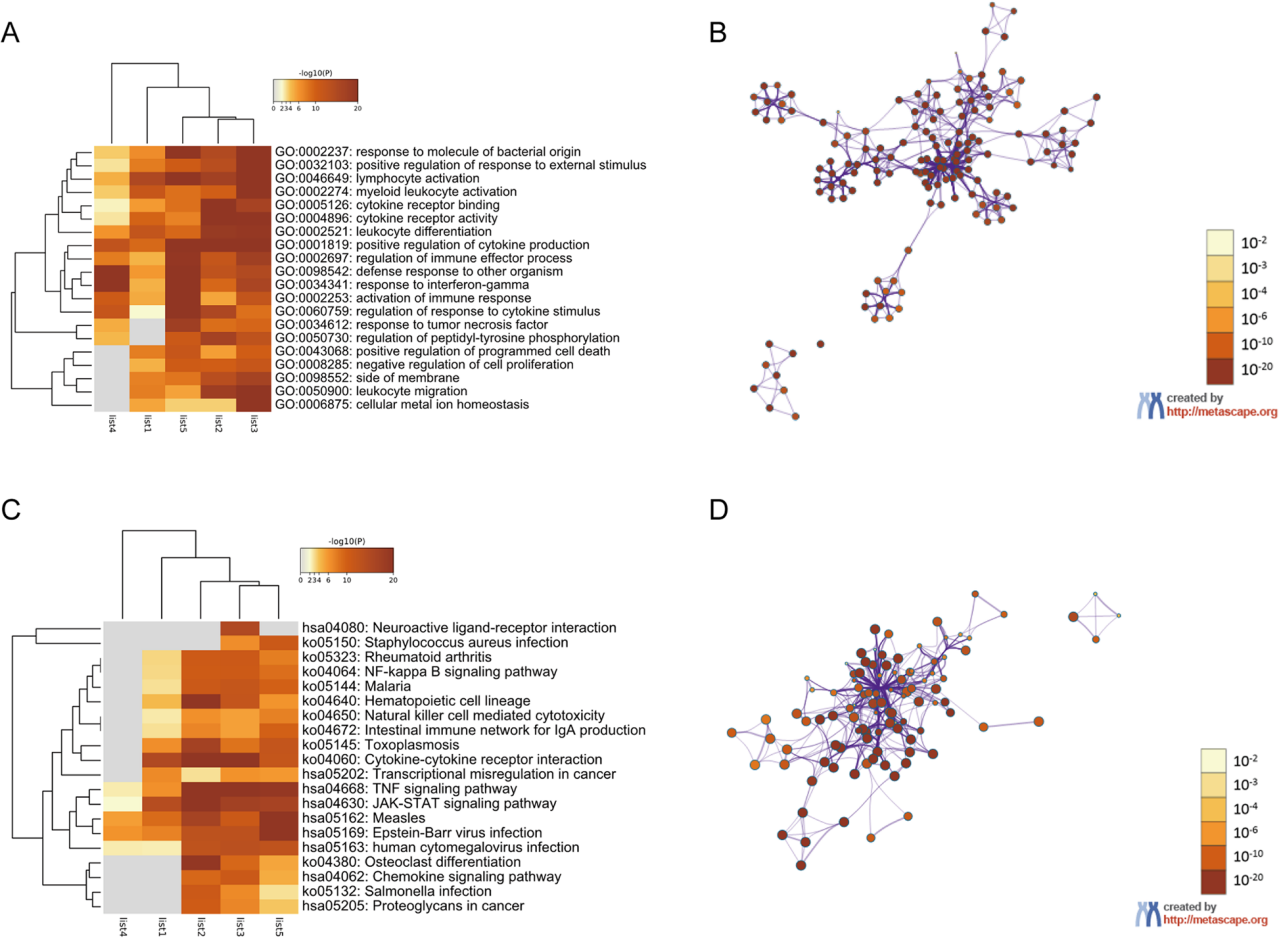
Enrichment analysis of immune-related gene lists in the DR2 group. **A**: Heatmap of enriched GO terms colored based on *p*-value. **B**: Network of enriched GO terms colored based on *p*-value, with terms containing more genes tending to have more significant *p*-values. **C**: Heatmap of enriched KEGG terms colored based on *p*-value. **D**: Network of enriched KEGG terms colored based on *p*-value

The top 20 GO enriched terms for the DR2 group are shown in Fig. 4, separated into biological process (17 items), molecular function (2 items), and cellular component (1 item) categories. For biological process, the enriched GO terms included 0002237 (response to molecule of bacterial origin), 0032103 (positive regulation of response to external stimulus), 0046649 (lymphocyte activation), 0002274 (myeloid leukocyte activation), 0002521 (leukocyte differentiation), 0001819 (positive regulation of cytokine production), 0002697 (regulation of immune effector process), 0098542 (defense response to other organism), 0034341 (response to interferon-gamma), 0002253 (activation of immune response), 0060759 (regulation of response to cytokine stimulus), 0034612 (response to tumor necrosis factor), 0050730 (regulation of peptidyl-tyrosine phosphorylation), 0043068 (positive regulation of programmed cell death), 0008285 (negative regulation of cell proliferation), 0050900 (leukocyte migration), and 0006875 (cellular metal ion homeostasis). For molecular function, there were two GO terms, namely 0005126 (cytokine receptor binding) and 0004896 (cytokine receptor activity). 0098552 (side of membrane) was the only enriched GO item in the cellular component category in DR2.

The top 20 KEGG pathways identified for the DR2 group are shown in Fig. 4 (C, D), including hsa04080 (Neuroactive ligand-receptor interaction), ko05150 (*Staphylococcus aureus* infection), ko05323 (Rheumatoid arthritis), ko04064 (NF-kappa B signaling pathway), ko05144 (Malaria), ko04640 (Hematopoietic cell lineage), ko04650 (Natural killer cell mediated cytotoxicity), ko04672 (Intestinal immune network for IgA production), ko05145 (Toxoplasmosis), ko04060 (Cytokine–cytokine receptor interaction), hsa05202 (Transcriptional misregulation in cancer), hsa04668 (TNF signaling pathway), hsa04630 (JAK-STAT signaling pathway), hsa05162 (Measles), hsa05169 (Epstein-Barr virus infection), hsa05163 (human cytomegalovirus infection), ko04380 (Osteoclast differentiation), hsa04062 (Chemokine signaling pathway), ko05132 (Salmonella infection), and hsa05205 (Proteoglycans in cancer).

**Fig. 5:**
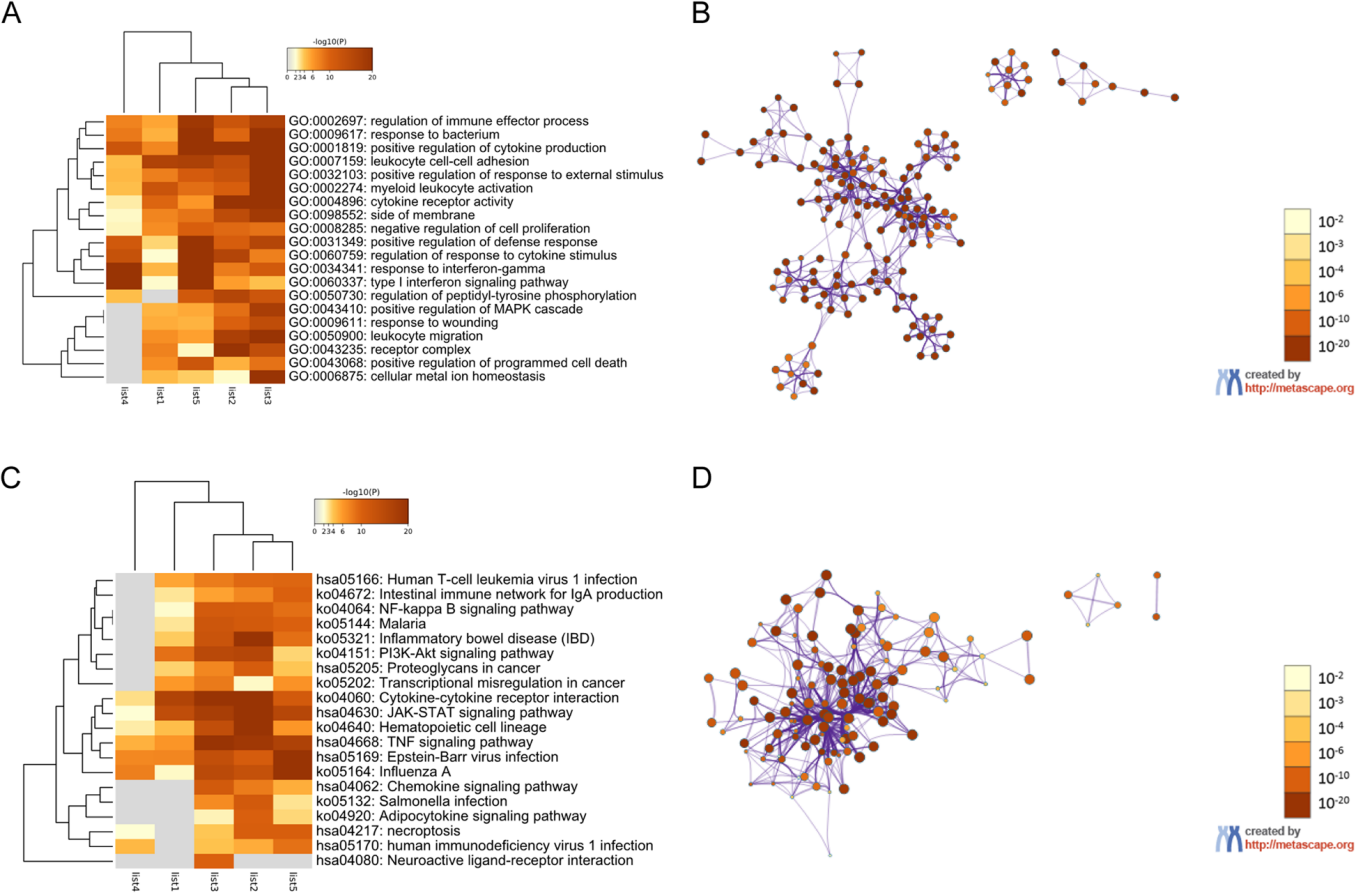
Enrichment analysis of immune-related gene lists in the DR1 group. **A**: Heatmap of enriched GO items colored based on *p*-value. **B**: Network of enriched GO terms colored based on *p*-value, where terms containing more genes tend to have more significant *p*-values. **C**: Heatmap of enriched KEGG items colored based on *p*-values. **D**: Network of enriched KEGG terms colored based on *p*-value

The 20 most enriched GO items for the DR1 group are shown in Fig. 5 (A, B). For biological process (17 items), these included 0002697 (regulation of immune effector process), 0009617 (response to bacterium), 0001819 (positive regulation of cytokine production), 0007159 (leukocyte cell-cell adhesion), 0032103 (positive regulation of response to external stimulus), 0002274 (myeloid leukocyte activation), 0008285 (negative regulation of cell proliferation), 0031349 (positive regulation of defense response), 0060759 (regulation of response to cytokine stimulus), 0034341 (response to interferon-gamma), 0060337 (type I interferon signaling pathway), 0050730 (regulation of peptidyl-tyrosine phosphorylation), 0043410 (positive regulation of MAPK cascade), 0009611 (response to wounding), 0050900 (leukocyte migration), 0043068 (positive regulation of programmed cell death), and 0006875 (cellular metal ion homeostasis). For molecular function, there was only one item, GO: 0004896 (cytokine receptor activity). For cellular component, there were two enriched GO items, 0098552 (side of membrane) and 0043235 (receptor complex).

The top 20 KEGG pathways in the DR1 group are shown in Fig. 5 (C, D), including hsa05166 (Human T-cell leukemia virus 1 infection), ko04672 (Intestinal immune network for lgA production), ko04064 (NF-kappa B signaling pathway), ko05144 (Malaria), ko05321 (Inflammatory bowel disease), ko04151 (PI3K-Akt signaling pathway), hsa05205 (Proteoglycans in cancer), ko05202 (Transcriptional misregulation in cancer), ko04060 (Cytokine–cytokine receptor interaction), hsa04630 (JAK-STAT signaling pathway), ko04640 (Hematopoietic cell lineage), hsa04668 (TNF signaling pathway), hsa05169 (Epstein-Barr virus infection), ko05164 (Influenza A), hsa04062 (Chemokine signaling pathway), ko05132 (Salmonella infection), ko04920 (Adipocytokine signaling pathway), hsa04217 (necroptosis), hsa05170 (human immunodeficiency virus 1 infection), and hsa04080 (Neuroactive ligand-receptor interaction).

### PPI analysis of immune-related gene sets

**Fig. 6:**
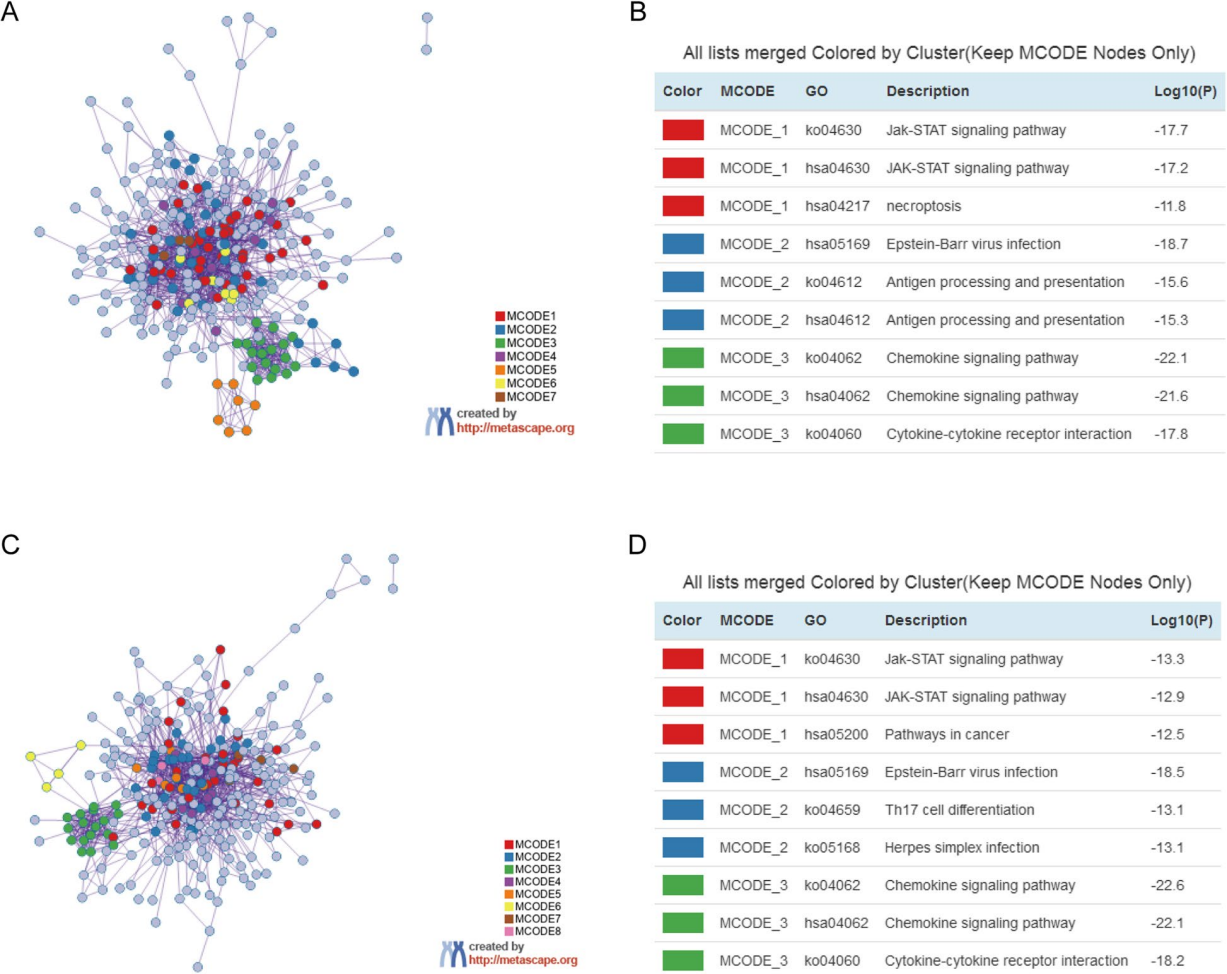
The PPI network and MCODE components for the two groups. **A**: PPI network for the DR2 group. **B**: The top three enriched MCODE components in DR2. **C**: PPI network for DR1. **D**: The top three enriched MCODE components

PPI analysis was performed using Metascape. The PPI network and the top three MCODE components were identified for DR2 (Fig. 6A, B) and DR1 (Fig. 6C, D) gene sets. The significant MCODE components were involved in the chemokine signaling pathway and cytokine–cytokine receptor interaction in the PPI network. The 17 genes with highest scores in both groups were CCR1, CCR7, CCL5, CCL20, CXCL1, CXCL3, CXCL8, CXCL9, CXCL10, FPR1, GNAI3, CCR4, CXCR6, C3AR1, LPAR1, C5AR1, and P2RY14.

### qRT-PCR analysis of diabetic mouse retinal tissue and HRMECs treated with HG

**Fig. 7:**
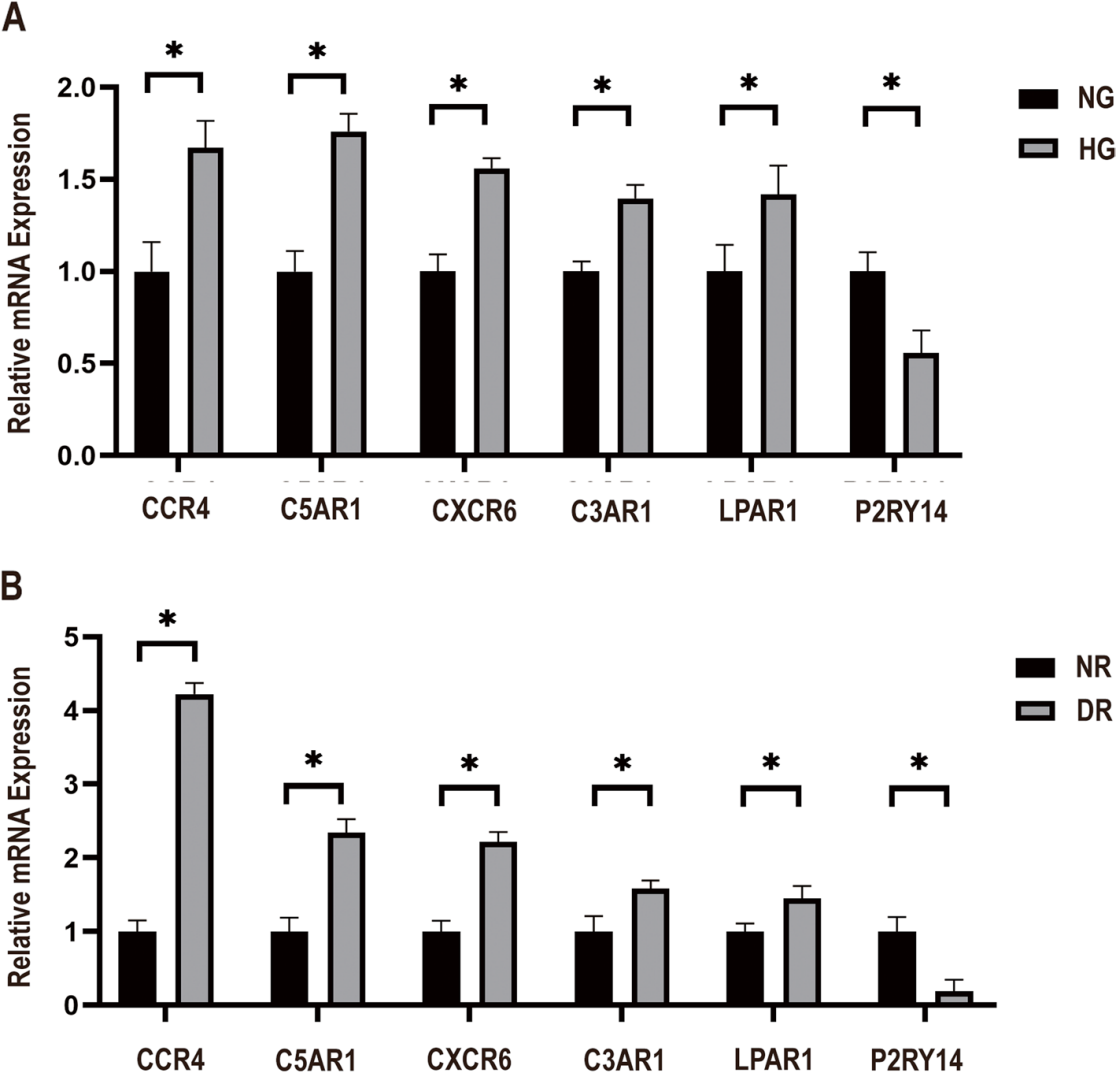
**A**: Differentially expressed genes in in HRMECs treated with HG and NG. **B**: Differentially expressed genes between DR and NR (*p* < 0.05)

qRT-PCR results indicated that the expression of CCR4, C5ar1, CXCR6, C3AR1, and LPAR1 was upregulated in both diabetic retina (DR) and HRMECs treated with HG, when compared to expression in the naive retina (NR) and NG. Notably, P2RY14 was significantly downregulated (Fig. 7. A, and B, *p* < 0.05).

## Discussion

In this study, we first separately analyzed immune-related gene expression in NVMs from PDR of both types in order to narrow down and identify potential genes implicated in PDR pathogenesis. The most significantly enriched pathways were mainly implicated in chemokine signaling and cytokine-cytokine receptor interactions. The current study provides better insight into the immune mechanisms underlying DR progression, with potential implications for diagnosis and treatment.

Chemokines are critical mediators of immune cell migration, with essential roles in immune surveillance, development, and inflammation. Chemokines exert their effects via transmembrane G protein-coupled receptors (GPCRs) present on a wide variety of cell types. Upon binding, conformational changes in trimeric G proteins trigger intracellular signaling pathways, promoting cellular movement and activation [17]. Based on the locations of conserved cysteine residues near the amino terminus, chemokines are divided into four subfamilies: C-C chemokine motif receptor (CCR), C-X-C chemokine motif receptor (CXCR), CX3CR, and XCR [18]. GPCRs regulate leukocyte trafficking and promote immune responses, mediating cell chemotaxis. GPCRs can be categorized into classical and chemokine subfamilies according to the ligand source. Classical GPCRs include formyl peptide receptors (FPR1, FPR2, and FPR3), platelet-activating factor receptor (PAFR), activated complement component 5 receptor (C5aR), and leukotriene B4 receptors (BLT1 and BLT2) [19].

In our study, 11 chemokines and their cognate receptors were identified as related to the pathogenesis of DR, including CCR1, CCR7, CCL5, CCL20, CXCL1, CXCL3, CXCL8, CXCL9, CXCL10, FPR1, and GNAI3.

CSF3, COL18A1, CXCR2, CCR1, FGF23, CXCL11, and IL13 were previously reported as related to PDR pathogenesis based on a Laplacian heat diffusion algorithm [20]. Therapeutic strategies targeting MIP1γ to inhibit CCR1-related signaling in retinal endothelial cells might have potential against DR progression [21]. CCR7 significantly enhanced neovascularization and the non-perfusion area in oxygen-induced retinopathy [22]. CCL5 could be measured in the blood, vitreous body, retina, aqueous humor, and tears of patients with DR [23]. C-C-chemokine receptor 6 is the only receptor interacting with CCL20. Treatment with CCL20-neutralizing antibodies or PG inhibits CCL20 expression, alleviating retinal degeneration and inflammation [24]. CCL20, CXCL2, and other core genes have been described as playing key roles in DR pathogenesis [25]. Activation of the P2X7R-NLRP3 pathway significantly increased the production of TNF-α, CXCL-1, CSF-1, IL-6, IL-1β, IL-18, and other pro-inflammatory cytokines in retinal microglia [26]. CXCL3, VEGF, CXCL5, and other inflammatory mediators were increased in DR and retinopathy of prematurity [27]. CXCL8 (also known as IL-8) is known to be elevated in the vitreous humor of patients with DR [28, 29]. Autocrine CXCL9 and CXCL10 signaling in retinal endothelial cells were enhanced in DR [30]. Recent research suggests that the level of CXCL10/IP-10 in normal vitreous humor was significantly higher than that in serum. Further, CXCL10/IP-10 levels in the vitreous humor of proliferative vitroretinopathy and PDR patients were much higher than that in patients with rhegmatogenous retinal detachment. CXCL10/IP-10 was also significantly upregulated in patients with active PDR compared to those with inactive disease [31]. CXCL10 derived from platelet-rich plasma exosomes can cause retinal endothelial injury, which was considerably alleviated by antagonizing CXCL10 with a neutralizing antibody [32]. UPARAR is a peptide inhibitor of the uPAR system, which could reverse the upregulation of uPAR, FPR1, and FPR2, thus slowing DR development [33]. The GαI1/3 protein plays a key role in vascular endothelial growth factor-induced endocytosis, signal transduction, and angiogenesis. High GαI1/3 protein expression was previously reported in the proliferative retinal tissue of PDR patients [34].

CCR4, CXCR6, C3AR1, LPAR1, C5AR1, and P2RY14 have been implicated in a number of eye diseases, but not in PDR. Upregulated CCR4 expression was previously observed in keratoconjunctivitis, glaucoma, and uveitis [35–37], in addition to its downregulation in dry eyes [38]. The IFN-γ and IL-17 expression of CD4^+^ T cells was significantly increased in patients with age-related macular degeneration. IFN-γ-expressing Th1 cells and IL-17-expressing Th17 cells could be selectively enriched based on the expression of surface CCR3^+^, CCR4^+^, CCR6^+^ [39]. CXCR6 was upregulated in the primary culture of orbital fibroblasts from patients with Graves’ orbitopathy, following treatment with pro-inflammatory cytokines IL-1β and TNF-α [40]. Furthermore, CXCR6 expression was highly confined to memory Th1 cells, which can be categorized into activated memory Th1 and Tc1 cells secreting IFN-γ [41]. A previous study reported that CD4^+^ and CXCR6^+^ cells were decreased in T1D patients [42]. A possible reason for the reduction in CD4^+^ cells expressing CXCR6, CXCR3, and CCR5 could be the selective recruitment of Th1 cells into the pancreas [43]. In human T cells, intracellular C5AR1 signaling induces ROS production through the mitochondria. ROS in turn trigger assembly of the NLRP3 (NACHT, leucine-rich repeat and pyrin domain-containing protein 3) inflammasome. Inflammasome formation initiates caspase-1-dependent IL-1β secretion, which promotes IFN-γ production and Th1 differentiation in an autocrine manner. Secreted C5a/C5a-Desarg interacts with surface-expressed C5AR2, which negatively controls NLRP3 activation through a currently undetermined mechanism [44]. Further, there is growing evidence that microglia-mediated inflammatory responses are associated with deleterious effects implicated in DR [7]. In fact, increased hypertrophic amoeba-like microglia were observed in the outer retina and subretinal space of human DR patients [45]. Overactivated amoeba-like microglia lead to a dysregulation of the complement system by upregulating the expression of activators C3, CFB, C1q, and C5AR1, while downregulating that of complement inhibitors CFH, CFI, CD46, and CD93 [46]. Subsequently, microglial overactivation establishes a pro-inflammatory environment conducive to further invasion of retinal microglia and exogenous monocyte infiltration [47]. The accumulation of subretinal microglia derived paracrine factors can trigger NLRP3 inflammasome activation in the retinal pigment epithelium [48]. Studies previously showed that complement may modulate the production of inflammatory factors and angiogenic factors via C5AR on Müller cells, which are implicated in DR pathogenesis [49]. C3AR1 is considered an injury-induced neuroinflammatory factor, whose interaction with IL-10 signaling and other immune-related pathways might be a major regulator of microglial activity and neuroinflammatory function [50]. In DBA/2 J mice, significant damage occurred with in the optic nerve head (ONH) prior to in other regions of the optic nerve [51]. At the same time point, the expression of C3AR1 in the ONH increased, with no increase observed in the retina [52]. In healthy brains, cell types other than the microglia exhibited low or no expression of C3AR1 [53]. The involvement of C3AR1/C5AR1 signaling in angiogenesis was reported on day 5 in ocular and retinal angiogenesis neonatal mouse models [54]. In our study, the sampled area included the area around the optic nipple. Previous works have shown that six G-coupled protein receptors (LPAR1–6) can be activated by lysophosphatidic acid (LPA). LPA and its receptors play vital roles in the central nervous system, cancer, and macular edema [55]. A link between LPA and retinopathy was previously demonstrated, as LPA1 and LPA2 expression were significantly increased in retinal ganglion cells after retinal ischemia in adult rats, leading to LPA1-mediated retinal ganglion cell death in preterm infants. In contrast, LPAR1–3 expression of retinal pigment epithelial cells promoted retinal healing. It was suggested that LPA exerts either a neuroprotective or neurodegenerative effect on the retina by binding to different LPA receptors on different cell types [56]. ATX, AGK, and LPA1 receptors are expressed in vascular endothelial cells and stromal cells within PDR epiretinal membranes. LPA-producing enzymes or LPA were shown to play a key role in the development of PDR and PVR [57]. The expression of P2Ry14 (purinergic receptor P2Y, G-protein coupled, 14) in the trabecular meshwork is higher than that of other purine receptors, suggesting that the protein product reduces IOP in monkeys, an observation that has not been further confirmed [58]. P2Ry14 was down regulated during both oxygen-induced pathologic neovascularization and physiological angiogenesis of the retina [59]. Purine receptor P2Y14 is highly expressed in collecting tube insertion cells and mediates renal aseptic inflammation [60].

He et al. previously suggested that differential ALDH2/SIRT1 expression might be responsible for the differences in DR severity between chronic inflammation-related T1 diabetes mellitus and T2 diabetes mellitus. Retinal IL-1 and IL-6 production in the T1 diabetes mellitus group was significantly increased compared to that in the T2 diabetes mellitus group [61]. In our study, we found no significantly enriched gene sets between type 1 and type 2 PDR groups. Li et al. previously reported that the prevalence of DR in diabetes patients was affected by diabetes duration, diabetic nephropathy occurrence, and regular DR screening. Diabetes type indirectly affected DR occurrence through its influence on diabetes duration and diabetic nephropathy occurrence [62].

In the present study, we found that six immune-related genes, namely, CCR4, CXCR6, C3AR1, LPAR1, C5AR1, and P2RY14, were upregulated based on bioinformatics analysis. CCR4, C5AR1, CXCR6, C3AR1, and LPAR1 expression was further confirmed via qRT-PCR, which was not the case for P2RY14, necessitating further study. Moreover, the upregulation of LPAR1 was found to have both neuroprotective and PDR promoting properties. Therefore, whether LPAR1 acts as a protective or pathogenic factor in PDR remains unclear.

## Conclusion

In summary, our findings provide insight into the molecular pathogenesis of DR, which may be of value for disease diagnosis and treatment. The roles of P2RY14 and LPAR1 in the pathogenesis of DR require further study. Our data provide a new idea for the diagnosis and treatment of DR in the future.

In our study, Nevertheless, the current study had some limitations, one being the limited number of neovascularization samples from patients with type 1 DR and the normal retina samples. We analyzed hub gene expression only via qRT-PCR, and it should be further validated via western blotting. Meanwhile, the alterations of immune-elated pathways might be explored in the future.

## Data Availability

The datasets analyzed during the current study are available in the “GSE102485”, (http://www.ncbi.nlm.nih.gov/geo/).

## Abbreviations

DR: Diabetic retinopathy
PDR: Proliferative diabetic retinopathy
NVMs: Neovascular membrane samples
HRMECs: Human retina microvascular endothelial cells
qRT-PCR: Quantitative Real Time Polymerase Chain Reaction
PPI: Protein-protein interaction
NV: Neovascularization
DEGs: Differentially expressed genes
FVMs: Fibrovascular membranes
PGF: Placental Growth Factor
GSEA: the Gene Set Enrichment Analysis
MSigDB: the Molecular Signatures Database
NES: Normalized enrichment scores
FDR: False discovery rate
GPCRs: G protein-coupled receptors
CCR: C-C cchemokine motif receptor
CXCR: C-X-C cchemokine motif receptor
FPR: Formyl peptide receptor
PAFR: Platelet-activating factor receptor
C5aR: Component 5 receptor
BLT: Leukotriene B4 receptor
PRP-Exos: Platelet-rich plasma exosomes
ONH: Optic nerve head
LPAR1–6: Six G-coupled protein receptors
LPA: Lysophosphatidic acid
ICS: Insertion cells
